# The relationship of weight change trajectory with medial temporal lobe atrophy in patients with mild Alzheimer’s disease: results from a cohort study

**DOI:** 10.1186/s13195-015-0098-1

**Published:** 2015-04-06

**Authors:** Erika Droogsma, Dieneke van Asselt, Hanneli Bieze, Nic Veeger, Peter Paul De Deyn

**Affiliations:** Department of Geriatric Medicine, Medical Centre Leeuwarden, Henri Dunantweg 2, 8934 AD Leeuwarden, the Netherlands; Department of Epidemiology, Medical Center Leeuwarden, Henri Dunantweg 2, 8934 AD Leeuwarden, the Netherlands; Department of Epidemiology, University Medical Center Groningen, University of Groningen, Hanzeplein 1, 9700 RB Groningen, the Netherlands; Department of Neurology and Alzheimer Research Center, University Medical Center Groningen, University of Groningen, Hanzeplein 1, 9700 RB Groningen, the Netherlands; Department of Neurology and Memory Clinic, ZNA and Laboratory of Neurochemistry and Behavior, Institute Born-Bunge, University of Antwerp, Campus Drie Eiken, Universiteitsplein 1, 2610 Wilrijk, Antwerp Belgium

## Abstract

**Introduction:**

Weight loss has been described in 20% to 45% of patients with Alzheimer’s disease (AD) and has been associated with adverse outcomes. Various mechanisms for weight loss in AD patients have been proposed, though none has been proven. This study aimed to elucidate a mechanism of weight loss in AD patients by examining the hypothesis that weight loss is associated with medial temporal lobe atrophy (MTA).

**Methods:**

Patients from the Frisian Alzheimer’s disease cohort study (a retrospective, longitudinal study of 576 community-dwelling AD patients) were included when a brain MRI was performed on which MTA could be assessed. To investigate the hypothesis that weight loss is associated with MTA, we investigated whether the trajectory of body weight change depends on the severity of MTA at the time of diagnosis (that is baseline). We hypothesized that patients with more severe MTA at baseline would have a lower body weight at baseline and a faster decrease in body weight during the course of the disease. The generalized linear mixed model (GLMM) was used to determine the relationship of weight change trajectory with MTA severity.

**Results:**

In total, 214 patients (median age 79 years, median MMSE 23, mean weight 73.9 kg) were included. Patients with moderate, severe or very severe MTA at baseline weighed 3.2 to 6.8 kg more than patients with no or mild MTA. During the 3.5 years, patients gained on average 1.7 kg in body weight, irrespective of the severity of their MTA at baseline.

**Conclusions:**

We found no evidence that MTA is associated with weight loss in AD patients. Moreover, contrary to what was expected, AD patients did not lose but gained weight during follow-up.

## Introduction

Various studies have investigated the relationship between body weight and Alzheimer’s disease (AD). On the one hand, being overweight has been associated with poorer cognitive function [1] and has been described as a risk factor for AD [2]. On the other hand, in 1907, Alois Alzheimer described weight loss in his first patient [3] and weight loss is currently recognized as a clinical feature of AD [4]. In the present study, we will focus on the relationship between weight loss and AD.

Weight loss has been described in approximately 20% to 45% of patients with AD [5-11] and has been associated with adverse outcomes such as an accelerated progression of AD [5,7,11,12], a higher rate of institutionalization [13] and increased mortality [14-16]. Various mechanisms of weight loss in AD patients have been proposed [5,17-19], though, none has been proven.

The regulation of body weight is complex and influenced by various factors such as appetite, feeding behavior and endocrine systems [20,21]. In addition, it is supposed that different brain areas are involved, one of which is the medial temporal lobe, possibly by influencing food intake and appetite [22-24]. Moreover, atrophy of the medial temporal lobe reflects changes in functional neuroanatomical networks that are involved in the regulation of body weight [22]. The medial temporal lobe is a site where AD pathology is typically present [25]. On magnetic resonance imaging (MRI), it shows atrophy in the earliest stages of the disease, which worsens as AD progresses [25]. Grundman *et al*. showed that medial temporal lobe atrophy (MTA) was associated with low body weight in AD patients [22]. Because of the cross-sectional design it was not possible to attribute cause and effect relations in this study, that is, weight loss may be a result of MTA or conversely, aggravate MTA [17,22]. If the latter is true, weight gain, for example by providing nutritional interventions, might prevent or slow MTA and possibly disease progression.

Few studies investigated the relationship between brain pathology and nutritional status in AD patients. The results of these studies are conflicting and none of the studies focused primarily on the relationship between MTA and nutritional status [26-28]. The aim of the present study was to elucidate a mechanism of weight loss in AD patients by examining the hypothesis that weight loss is associated with MTA.

## Methods

### Setting

This study was conducted with data from the Frisian Alzheimer’s disease cohort study, a retrospective, longitudinal study of the long-term course of 576 AD patients seen at a large memory clinic in the north of the Netherlands. Patients were evaluated by a physician and a specialized geriatric nurse who performed a comprehensive geriatric assessment (CGA) [29]. When a diagnosis of AD could not be established based on the CGA and cognitive screening tests, additional tests were ordered, including brain imaging, with MRI as the preferred imaging technique [30,31]. When the diagnosis of probable or possible AD (according to the criteria of the National Institute of Neurological and Communicative Diseases - Alzheimer’s Disease and Related Disorder Association (NINCDS-ADRDA)) [4] was established, patients were offered pharmacological (that is, cholinesterase inhibitors (ChEIs) and/or memantine) and non-pharmacological interventions (that is case management, respite care, meals at home services). Yearly outpatient visits were scheduled to evaluate the overall condition of the patient, including body weight, and the effect of the interventions. Outpatient visits ended when pharmacological treatment was terminated or in the case of nursing home admission. The total number of outpatient visits for patients from the Frisian Alzheimer’s disease cohort study ranged from one to eleven, the median number was three (that is 1.5 years) (25th to 75th percentile 2 to 5).

### Participants and study design

Patients included in the Frisian Alzheimer’s disease cohort study visited the memory clinic between 2002 to 2012, were 65 years or older, lived at home or in residential care at the time of diagnosis and started with a ChEI. Patients were included in the present study when they had a baseline assessment, at least one follow-up assessment and a MRI of the brain (performed up to 6 months prior to AD diagnosis) on which MTA could be assessed. To examine the hypothesis that weight loss is associated with MTA, we investigated whether the trajectory of body weight change depends on the severity of MTA at the time of diagnosis (that is baseline). We hypothesized that patients with more severe MTA at baseline would have a lower body weight at baseline and a faster decrease in body weight during the course of the disease. This study, for which informed consent was not required, was approved by the local ethics committee of the Medical Center Leeuwarden. Informed consent was not required because this was a retrospective chart study in which the anonymity of the patients was guaranteed.

### Measurements

#### Sociodemographic characteristics

Age, gender, social status, use of informal and of professional care (that is household help, meals at home services) were recorded. Comorbidity was evaluated by the cumulative illness rating scale (CIRS) with total scores ranging from 0 (no impairment) to 56 (extremely severe impairment) [32]. AD as the index disease was not included in the CIRS score. The number of medications beside the ChEI was recorded. Polypharmacy was defined as use of four or more medications beside the ChEI.

#### Cognitive functioning

Cognitive functioning was assessed by the mini mental state examination (MMSE) [33] and the clock-drawing test (CDT). The CDT was scored according the scoring system of Shulman *et al.* in 1993, in which the total score ranges from 1 to 6, and a score of 3 or more indicates cognitive impairment [34].

#### Behavioral and psychological symptoms (BPS) of dementia

Based on self-reported patient and caregiver information, we recorded whether BPS were present or absent. Since BPS were not operationalized with a measurement instrument, it was not possible to report the severity, nature or frequency of BPS.

#### Type and dosage of ChEI

At each outpatient visit, type and dosage of ChEI, and if applicable memantine, were recorded. At our memory clinic, galantamine retard is the first choice of treatment for patients with mild to moderate AD. The retard form of galantamine has been prescribed since 2005. Before 2005, galantamine was given twice daily. The dose is gradually increased from 8 milligram (mg) per day to 24 mg per day in 8 weeks.

#### Nutritional status

Body weight (kg), body mass index (BMI), self-reported weight loss, appetite and use of oral nutritional supplements (ONS) were recorded. Self-reported weight loss, appetite and use of ONS were recorded based on patient and caregiver information.

#### Medial temporal lobe atrophy (MTA)

Brain MRIs were obtained with a Philips 3.0 Tesla MRI scanner (Philips, Eindhoven, the Netherlands). MTA was assessed on coronal three-dimensional gradient T1-weighted MRI sequences. MTA was rated using a validated 5-point visual rating scale, based on the evaluation of the width of the choroidal fissure, the width of the temporal horn and the hippocampal height [35,36]. The severity of MTA was scored from 0 (no atrophy) to 4 (very severe atrophy) [35]. According to the instructions of the visual rating scale, both left and right MTA were examined [37,38]. MTA was scored independently by two raters (ED and HB, both research fellows) who were trained in rating MTA by an experienced neurologist. The agreement between the two raters was measured by calculating the kappa value [39]. Disagreement between ED and HB was resolved by discussion with a third rater (DA).

### Statistical analysis

Data were analyzed with the Statistical Package for the Social Sciences (SPSS) 16.0 (SPSS Inc., Chicago, IL, USA) and Statistical Analysis Software (SAS) 9.2 (SAS Institute Inc., Cary, NC, USA). Hypotheses were two-tailed tested. A probability (*P*) value of less than 0.05 was considered statistically significant. Descriptive statistics are presented as mean ± standard deviation (SD) for normally distributed variables. For skewed distributed variables, median and 25th to 75th percentiles are given. We used the Kolmogorov-Smirnov test to establish the distribution of the variable. Number and proportion are given for categorical variables.

To investigate whether the trajectory of body weight change depends on the severity of MTA at baseline, the trajectory of body weight change was compared between MTA score groups, using the generalized linear mixed model (GLMM) [40,41]. The GLMM has been developed for the analysis of longitudinal, dependent data and provides an estimate of change in the dependent variable (that is body weight) over time. The GLMM analyses were performed with data from patients with a baseline assessment and at least one follow-up assessment. Otherwise, it is not possible to describe a change in body weight over time. Scores of left and right MTA were used separately for analyses. From the sixth (left MTA), respectively the fifth (right MTA) measurement moment, it was not possible to give a reliable estimate of change in body weight due to the small number of remaining patients at that moment. Therefore, the GLMM analyses were performed on the first five measurement moments (a period of 3.5 years) for left MTA scores, respectively on the first four measurements (a period of 2.5 years) for right MTA scores.

The relationship of left MTA and right MTA with the trajectory of weight was analyzed in a multivariate GLMM, including potential confounders. The multivariate GLMM analyses were performed according the backward-method with weight as the primary outcome (that is the dependent variable). MTA group, time (that is the number of measurement moments) and the potential confounders were used as independent variables. Potential confounders were baseline variables associated with the course of weight in univariate GLMM analysis, it involved: gender (*P* <0.001), social status (*P* = 0.048), informal care (*P* = 0.003) and self-reported weight loss (*P* <0.001). To correct for potential interactions, we investigated whether there were interactions of left MTA and right MTA score with time or with the four potential confounders.

Various baseline characteristics were compared between patients with a MTA score of 0 or 1 versus a MTA score of 2, 3 or 4. The independent sample *t* test was performed to compare normally distributed variables. We employed the Mann-Whitney *U* test to compare skewed distributed variables. Pearson chi-square or Fisher’s exact test were used to compare categorical variables.

## Results

### Patient characteristics at baseline

Two hundred and fourteen patients were included. At baseline, median age was 79 years (25th to 75th percentile 75.0 to 82.0), median MMSE score 23 (25th to 75th percentile 20.0 to 25.0) and mean weight 73.8 ± 12.0 kg. More than half of the patients had mild AD and none of the patients had severe AD. Almost one in every six patients reported weight loss (14.4%) and thirteen patients (7.1%) had a poor appetite (Table 1). An additional table summarizes the number of patients who experienced weight loss, weight gain or no change in weight (Table 2).

**Table 1:** **Patient characteristics at baseline**

**Age** (year): n, median [25^th^ -75^th^ percentile]	214, 79 [75 - 82]
**Women**, n (%)	132 (61.7)
**Social status**	
alone, n (%)	93 (44.9)
with partner, n (%)	108 (52.2)
other*, n (%)	6 (2.9)
**CIRS** (score): n, median [25^th^ -75^th^ percentile]	214, 6.0 [4.0- 8.0]
**Number of medications beside ChEI**	
≤4, n (%)	100 (46.9)
>4 (polypharmacy), n (%)	113 (53.1)
**Use of informal care,** n (%)	182 (85.4)
**Use of professional care**, n (%)	99 (46.9)
**MMSE** (score): n, median [25^th^ -75^th^ percentile]	212, 23 [20 - 25]
MMSE score ≤24, n (%)	138 (65.1)
MMSE >26, n (%)	33 (15.6)
Mild AD (MMSE 21 - 26), n (%)	126 (59.4)
Moderate AD (MMSE 10 - 20), n (%)	53 (25.0)
Moderate severe AD (MMSE 10 - 14), n (%)	3 (1.4)
Severe AD (MMSE <10), n (%)	0 (0)
**Clock-drawing test** (score): n, median [25^th^ -75^th^ percentile]	193, 3.0 [2.0 - 4.5]
Clock-drawing test score ≥3, n (%)	140 (72.5)
**Presence of BPS,** n (%)	51 (24.4)
**ChEI which was started**	
galantamine, n (%)	209 (97.7)
rivastigmine, n (%)	5 (2.3)
**Weight** (kg): n, mean ± SD	214, 73.8 ± 12.0
**BMI** (weight/(height)^2^): n, median [25^th^ -75^th^ percentile]	203, 25.8 [23.5 - 28.6]
**Use of ONS**, n (%)	1 (0.5)
**Appetite**	
good, n (%)	170 (92.9)
poor, n (%)	13 (7.1)
**Self-reported weight loss,** n (%)	28 (14.4)
**Left MTA score**	
0 (no atrophy), n (%)	1 (0.5)
1 (mild atrophy), n (%)	15 (7.0)
2 (moderate atrophy), n (%)	111 (51.9)
3 (severe atrophy), n (%)	65 (30.4)
4 (very severe atrophy), n (%)	22 (10.3)
**Right MTA score**	
0 (no atrophy), n (%)	2 (0.9)
1 (mild atrophy), n (%)	15 (7.0)
2 (moderate atrophy), n (%)	108 (50.5)
3 (severe atrophy), n (%)	74 (34.6)
4 (very severe atrophy), n (%)	15 (7.0)

*Other, that is: with son or daughter, brother or sister. n, number of patients; CIRS, cumulative illness rating scale; ChEI, cholinesterase inhibitor; MMSE, mini mental state examination; AD, Alzheimer’s disease; BPS, behavioral and psychological symptoms; SD, standard deviation; BMI, body mass index; ONS, oral nutritional supplement; MTA, medial temporal lobe atrophy.

**Table 2:** **Number of patients with weight loss, weight gain or no change in weight over time**

	**No change in weight: n, (%)**	**Weight loss: n, (%) mean weigh loss (kg) ± SD**	**Weight gain: n, (%) mean weight gain (kg) ± SD**
Between baseline	6 (3.1)	101 (52.6)	85 (44.3)
and 6 months		−2.9 ± 2.7	2.5 ± 2.3
Between 6 months	7 (5.0)	53 (37.6)	81 (57.4)
and 18 months		−2.7 ± 2.2	2.7 ± 2.4
Between 18 months	4 (3.8)	41 (38.7)	61 (57.5)
and 30 months		−2.1 ± 1.4	2.6 ± 1.8
Between 30 months	3 (4.1)	28 (38.4)	42 (57.5)
and 42 months		−3.1 ± 3.9	3.6 ± 4.6

n, number of patients; SD, standard deviation.

### Medial temporal lobe atrophy (MTA)

The MRI scans were performed median 20.5 days (25th to 75th percentile 45.0 to 8.0) before the diagnosis AD was made. Three patients (1.4%) had a MTA score of 0 (Table 1), they were clustered with patients with a MTA score of 1, representing patients with no or mild MTA. In 75.4% of the patients, the MTA of the left side was the same as on the right side. The agreement between the two raters was fair to good for left MTA (Cohen’s kappa 0.66) and fair for right MTA (Cohen’s kappa 0.60) [39,42].

### Relationship of left MTA with the trajectory of weight change

**Figure 1:**
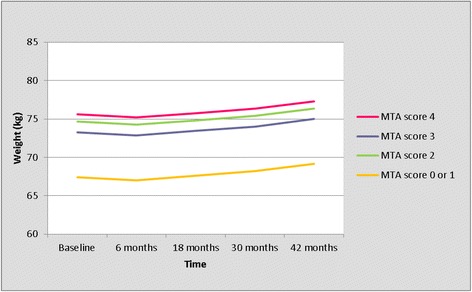
**Trajectory of weight per left MTA score (results from the univariate GLMM analyses).** GLMM, generalized linear mixed model; MTA, medial temporal lobe atrophy.

**Figure 2:**
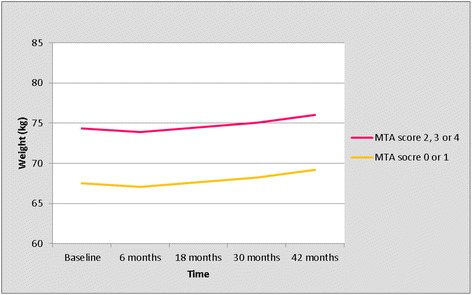
**Trajectory of weight; left MTA score of 0 or 1 versus 2, 3 or 4 (results from the univariate GLMM analyses).** GLMM, generalized linear mixed model; MTA, medial temporal lobe atrophy.

The trajectory of weight change for left MTA score is presented in Table 3 and Figure 1. During the first 6 months, body weight decreased with 0.4 kg in all MTA groups (Table 3, Figure 1). Thereafter, it increased gradually with 2.1 kg in the subsequent 3 years (Figure 1, Table 3). Overall, all patients gained on average 1.7 kg in body weight during 3.5 years (Table 3, Figure 1). Figure 1 and Table 3 show an obvious difference in body weight between patients with a MTA score of 0 or 1, versus patients with a MTA score of 2, 3 or 4 (Table 3, Figure 1). Because of this clear difference with a highly comparable trajectory of weight in patients with MTA score 2, 3 and 4, we compared the trajectory of weight between patients with a MTA score of 0 or 1, versus patients with a MTA score of 2, 3 or 4 (Table 4, Figure 2). Patients with MTA 0 or 1 weighed 6.8 kg less than patients with MTA 2, 3, 4 at every moment during follow-up, independent of potential confounders (*P* = 0.001). There were no interactions of the left MTA score with time or with the four potential confounders. As shown in Table 5, patients with MTA 0 or 1 were younger and had a higher MMSE score at baseline compared to patients with a MTA score of 2, 3 or 4 (Table 5).

**Table 3:** **Trajectory of weight per left MTA score (results from the univariate GLMM analyses)**

	**n**	**MTA score 0 or 1**	**MTA score 2**	**MTA score 3**	**MTA score 4**
		**mean weight (95% CI)**	**mean weight (95% CI)**	**mean weight (95% CI)**	**mean weight (95% CI)**
Baseline	214	67.44 (61.87 - 73.01)	74.67 (63.17 - 86.17)	73.27 (61.51 - 85.03)	75.59 (62.71 - 88.47)
6 months	194	67.02 (60.97 - 73.08)	74.25 (62.26 - 86.25)	72.85 (60.60 - 85.11)	75.17 (61.80 - 88,54)
18 months	160	67.60 (61.32 - 73.88)	74.83 (62.62 - 87.04)	73.43 (60.96 - 85.91)	75.75 (62.16 - 89.34)
30 months	116	68.18 (61.80 - 74.57)	75.42 (63.09 - 87,74)	74.01 (61.43 - 86.60)	76.33 (62.63 - 90.04)
42 months	79	69.15 (62.18 - 76.12)	76.38 (63.48 - 89.28)	74.98 (61.81 - 88.14)	77.30 (63.01 - 91.58)

MTA, medial temporal lobe atrophy; GLMM, generalized linear mixed model; n, number of remaining patients; CI, confidence interval.

**Table 4:** **Trajectory of weight: left MTA 0 or 1 versus 2, 3 or 4 (results from the univariate GLMM analyses)**

	**n**	**MTA score 0 or 1**	**MTA score 0 or 1**	**MTA score 2, 3, 4**	**MTA score 2, 3, 4**
		**mean weight (95% CI)**	**∆ Weight**	**mean weight (95% CI)**	**∆ Weight**
Baseline	214	67.49 (61.91 - 73.07)		74.31 (62.95 - 85.67)	
6 months	194	67.07 (61.00 - 73.14)	−0.42	73.89 (62.05 - 85.74)	−0.42
18 months	160	67.65 (61.36 - 73.94)	0.58	74.47 (62.40 - 86.54)	0.58
30 moths	116	68.24 (61.84 - 74.63)	0.59	75.06 (62.88 - 87.23)	0.59
42 months	79	69.19 (62.21 - 76.17)	0.95	76.01 (63.26 - 88.77)	0.95

MTA, medial temporal lobe atrophy; GLMM, generalized linear mixed model; n, number of remaining patients; CI, confidence interval.

**Table 5:** **Patient characteristics at baseline: left MTA score of 0 or 1 versus 2, 3 or 4**

	**MTA score 0 or 1**	**MTA score 2, 3, 4**	***P***
**Age** (year): n, median [25^th^ -75^th^ percentile]	16, 75.0 [72.0 - 78.8]	198, 79.0 [75.8 - 82.0]	0.003^b**^
**Women**, n (%)	11 (68.8)	121 (61.1)	ns^c^
**Social status**			
alone, n (%)	10 (62.5)	83 (43.5)	ns^c^
with partner, n (%)	5 (31.3)	103 (53.9)	
other*, n (%)	1 (6.3)	5 (2.6)	
**CIRS** (score): n, median [25^th^ -75^th^ percentile]	16, 5.0 [4.0 - 6.0]	198, 6.0 [4.0 - 8.0]	ns^b^
**Polypharmacy,** n (%)	8 (50.0)	105 (53.3)	ns^c^
**Use of informal care,** n (%)	13 (81.3)	169 (85.8)	ns^d^
**Use of professional care**, n (%)	6 (37.5)	93 (47.7)	ns^c^
**MMSE** (score): n, median [25^th^ -75^th^ percentile]	16, 25.0 [23.0 - 26.8]	196, 23.0 [20.0 - 15.0]	0.017^b^**
**Clock-drawing test** (score): n, median [25^th^ -75^th^ percentile]	15, 3.0 [1.0 - 4.0]	178, 3.0 [2.0 -5.0]	ns^b^
**Presence of BPS,** n (%)	5 (31.3)	46 (23.8)	ns^d^
**Weight** (kg): n, mean ± SD	16, 68 ± 12	198, 74 ± 12	ns^a^
**BMI** (weight/(height)^2^): n, median [25^th^ -75^th^ percentile]	16, 25.7 [21.1 - 27.0]	187, 25.9 [23.5 - 29.1]	ns^b^
**Use of ONS**, n (%)	0 (0.0)	1 (0.8)	ns^d^
**Appetite**			
good, n (%)	14 (87.5)	156 (93.4)	ns^d^
poor, n (%)	2 (12.5)	11 (6.6)	
**Self-reported weight loss,** n (%)	2 (12.5)	26 (14.6)	ns^d^

*Other, that is: with son or daughter, brother or sister; **significant, that is *P* <0.05. ^a^Independent sample *t* test; ^b^Mann-Whitney *U* test; ^c^Pearson chi-square test; ^d^Fisher’s exact.

MTA, medial temporal lobe atrophy; *P*, probability; n, number of patients; ns, not significant; CIRS, cumulative illness rating scale; MMSE, mini mental state examination; BPS, behavioral and psychological symptoms; SD, standard deviation; BMI, body mass index; ONS, oral nutritional supplement.

### Relationship of right MTA with the trajectory of weight change

**Figure 3:**
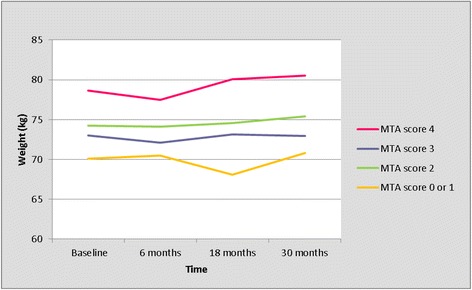
**Trajectory of weight per right MTA score (results from the univariate GLMM analyses).** GLMM, generalized linear mixed model; MTA, medial temporal lobe atrophy.

**Figure 4:**
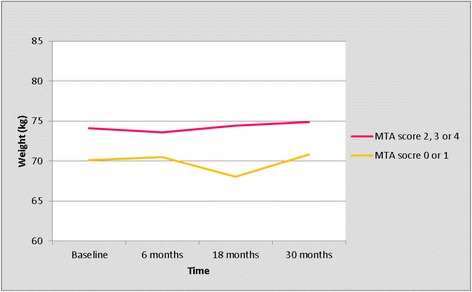
**Trajectory of weight; right MTA score 0 or 1 versus 2, 3 or 4 (results from the univariate GLMM analyses).** GLMM, generalized linear mixed model; MTA, medial temporal lobe atrophy.

The trajectory of weight per right MTA score is presented in Table 6 and Figure 3. As for the left MTA, we compared the trajectory of weight between patients with a MTA score of 0 or 1, versus patients with a MTA score of 2, 3 or 4 (Table 7, Figure 4). Patients with MTA 0 or 1 weighed less than patients with MTA 2, 3, 4 at any moment during follow-up. In this, an interaction with time (*P* = 0.001) was observed, depending on the moment during follow-up, patients with MTA 0 or 1 weighed between 3.2 kg (at 6 months) and 6.4 kg (at 18 months) less than patients with MTA 2, 3, 4 (Table 7, Figure 4). Overall, all patients gained on average 0.7 kg in body weight after 2.5 years (Table 7). As for the left MTA, we compared the baseline characteristics between patients with MTA 0 or 1, versus patients with a MTA score of 2, 3 or 4 (Table 8). Patients with MTA 0 or 1 were younger, had a higher MMSE score and used informal care less often at baseline compared to patients with a MTA score of 2, 3 or 4 (Table 8).

**Table 6:** **Trajectory of weight per right MTA score (results from the univariate GLMM analyses)**

	**n**	**MTA score 0 or 1**	**MTA score 2**	**MTA score 3**	**MTA score 4**
		**mean weight (95% CI)**	**mean weight (95% CI)**	**mean weight (95% CI)**	**mean weight (95% CI)**
Baseline	214	70.12 (64.46 - 75.78)	74.23 (62.49 - 85.98)	73.03 (61.10 - 84.97)	78.62 (64.70 - 92.54)
6 months	194	70.48 (63.13 - 77.82)	74.11 (58.86 - 89.36)	72.13 (56.63 - 87.63)	77.51 (59.43 - 95.60)
18 months	160	68.06 (59.85 - 76.27)	74.56 (57.52 - 91,59)	73.12 (55.83 - 90.42)	80.07 (59.90 - 100.23)
30 months	116	70.79 (62.24 - 79.33)	75.43 (57.70 - 93.16)	72.94 (54.92 - 90.95)	80.50 (59.22 - 101.77)

MTA, medial temporal lobe atrophy; GLMM, generalized linear mixed model; n, number of remaining patients; CI, confidence interval.

**Table 7:** **Trajectory of weight: right MTA 0 or 1 versus 2, 3 or 4 (results from the univariate GLMM analyses)**

	**n**	**MTA score 0 or 1**	**MTA score 0 or 1**	**MTA score 2, 3, 4**	**MTA score 2, 3, 4**
		**mean weight (95% CI)**	**∆ Weight**	**mean weight (95% CI)**	**∆ Weight**
Baseline	214	70.12 (64.42 - 75.81)		74.12 (62.49- 85.75)	
6 months	194	70.48 (63.09 - 77.87)	0.36	73.62 (58.53 - 88.72)	−0.5
18 months	160	68.06 (59.80 - 76.32)	−2.42	74.43 (57.57- 91.30)	0.81
30 moths	116	70.79 (62.17 - 79.40)	2.74	74.88 (57.28 - 92.47)	0.45

MTA, medial temporal lobe atrophy; GLMM, generalized linear mixed model; n, number of remaining patients; CI, confidence interval.

**Table 8:** **Patient characteristics at baseline: right MTA score of 0 or 1 versus 2, 3 or 4**

	**MTA score 0 or 1**	**MTA score 2, 3, 4**	***P***
**Age** (year): n, median [25^th^ -75^th^ percentile]	17, 73.0 [72.0 - 79.0]	197, 79.0 [76.0 - 82.0]	0.003^b^**
**Women**, n (%)	9 (52.9)	123 (62.4)	ns^c^
**Social status**			
alone, n (%)	8 (47.1)	85 (44.7)	ns^c^
with partner, n (%)	9 (52.9)	99 (52.1)	
other*, n (%)	0 (0. 0)	6 (3.2)	
**CIRS** (score): n, median [25^th^ -75^th^ percentile]	17, 5.0 [4.0 - 6.5]	197, 6.0 [4.0 - 8.0]	ns^b^
**Polypharmacy,** n (%)	7 (41.2)	106 (54.1)	ns^c^
**Use of informal care,** n (%)	11 (64.7)	171 (87.2)	0.022^d^**
**Use of professional care**, n (%)	5 (29.4)	94 (48.5)	ns^c^
**MMSE** (score): n, median [25^th^ -75^th^ percentile]	17, 25.0 [22.0 - 27.5]	195, 23.0 [20.0 - 25.0]	0.026^b^**
**Clock-drawing test** (score): n, median [25^th^ -75^th^ percentile]	16, 3.0 [1.0 - 4.0]	177, 3.0 [2.0 - 5.0]	ns^b^
**Presence of BPS,** n (%)	3 (17.7)	48 (25.0)	ns^d^
**Weight** (kg): n, mean ± SD	17, 70 ± 11	197, 74 ± 12	ns^a^
**BMI** (weight/(height)^2^): n, median [25^th^ -75^th^ percentile]	15, 25.6 [21.5 - 27.0]	188, 25.9 [23.5 - 28.9]	ns^b^
**Use of ONS**, n (%)	0 (0.0)	1 (0.6)	ns^d^
**Appetite**			
good, n (%)	16 (100)	154 (92.2)	ns^d^
poor, n (%)	0 (0.0)	13 (7.8)	
**Self reported weight loss,** n (%)	0 (0.0)	28 (15.8)	ns^d^

*Other, that is: with son or daughter, brother or sister; **significant, that is *P* <0.05. ^a^Independent sample *t* test; ^b^Mann-Whitney *U* test; ^c^Pearson chi-square test; ^d^Fisher’s exact.

MTA, medial temporal lobe atrophy; *P*, probability; n, number of patients; ns, not significant; CIRS, cumulative illness rating scale; MMSE, mini mental state examination; BPS, behavioral and psychological symptoms; SD, standard deviation; BMI, body mass index; ONS, oral nutritional supplement.

## Discussion

The aim of the present study was to elucidate a mechanism of weight loss in AD patients. Contrary to what was expected, AD patients in our population did not lose, but gained weight during the 3.5 years of follow-up. Recent studies have reported similar results [43,44]. Secher *et al*. showed that community-dwelling patients with moderate AD did not lose weight during 4 years of follow-up [43], Gu *et al*. showed that after the clinical onset of AD, BMI increased [44]. How can the increase in body weight be explained?

The number of community-dwelling AD patients with weight loss described in the literature, varies between 20% and 45% [5-10]. The highest numbers are reported in studies from the pre-ChEI era [5,6,9], and recent studies showed a decreased risk of weight loss in AD patients treated with a ChEI compared to untreated patients [7,8,45,46]. In these studies, ChEIs appeared to protect against weight loss. Therefore, the weight gain in our cohort might be explained by the use of a ChEI.

It could be that weight loss in AD patients is currently less frequently observed due to the increased quality of care of home-dwelling AD patients. In the past decade, it is not just the pharmacological treatment that has changed the management of AD. Drugs are given in addition to multiple non-pharmacological interventions, including dietary advice and provision of meals at home services [31,47]. Gu *et al*. showed that the BMI of AD patients declined up to the clinical onset of AD. After clinical onset, there was no decrease of BMI, which even increased, possibly because care was arranged after the diagnosis of AD [44]. We postulate that weight loss in AD patients could be regarded as a marker for the quality of care for AD patients, rather than a marker for the severity of AD. This is underpinned by our finding that the severity of AD, measured by the severity of MTA, was not related to the trajectory of weight.

Contrary to what was expected, there was no difference in body weight between patients with moderate, severe or very severe MTA, neither at the time of diagnosis, nor during the course of the disease. Moreover, during follow-up, a time period in which the severity of MTA is expected to increase, patients did not lose but gained weight. Therefore, we reject the hypothesis that weight loss is associated with MTA. As far we know, four other studies investigated the relation of brain pathology with nutritional status in AD patients [22,26-28]. Contrary to our finding, Grundman *et al*. showed that MTA was associated with low body weight in AD patients [22]. In addition, Burns *et al*. showed that a higher BMI was associated with less brain atrophy [27]. This association, however, was modest [27]. Hu *et al*. found no association between a low BMI and the medial temporal lobe [26], which is in line with our finding that there was no difference in body weight between patients with moderate, severe or very severe MTA. Ho *et al*. reported that more severe hippocampal atrophy was associated with a higher BMI in patients with mild AD [28], which confirms our result that patients with moderate, severe or very severe MTA weighed more than patients with no or mild MTA. These results also underline the findings of Gustafson *et al*. [48] and Ward *et al*. [49]. Gustafson *et al*. investigated the longitudinal relationship between BMI and MTA in a cohort of middle-aged women during 24 years of follow-up [48]. The average BMI of women who developed MTA was higher at all examinations than women who did not develop MTA [48]. In the same cohort, a higher BMI was associated with a higher incidence of dementia, particularly AD [2]. It is suggested that this latter relationship could be explained by the fact that being overweight is a risk factor for hypertension, type 2 diabetes, and cardiovascular diseases, all of which have been shown to increase the risk of AD [50]. Though, it is also possible that being overweight increases the risk of AD by directly affecting the neurodegenerative process in the brain [50]. Ward *et al*. performed a cross-sectional study to investigate the relationship between BMI and brain atrophy in middle-aged (40 to 66 years) adults [49]. A higher BMI was associated with more severe brain atrophy, though BMI was not associated with cognitive function [49].

It must be taken into consideration that comparison of our study with the aforementioned studies has to be performed cautiously, because of differences in patient characteristics and study methodology. For example, patients in the study of Grundman *et al*. and Burns *et al*. were not treated with a ChEI. In addition, the severity of AD varied with mean MMSE score in the study of Grundman *et al*. of 19 [18], in the study of Burns *et al*. 26 [23], versus a median MMSE of 23 in our cohort. Not all patients in the study of Gustafson *et al*. and Ward *et al*. were diagnosed with AD at baseline [48,49]. Moreover, the way in which brain pathology was measured differed. In the present study, brain atrophy was measured with MRI, Hu *et al*. investigated brain glucose metabolism by positron emission tomography (PET) and [^18^F]fluordeoxyglucose (FDG) [26], Ho *et al*. applied an automated hippocampal mapping method to measure hippocampal volume [28] and Grundman *et al*. performed morphometric analysis to assess the severity of MTA [22], while we used a visual rating scale to assess MTA. These differences may have contributed to the conflicting results. The conflicting results might also reflect variation in sample sizes, ranging from 27 [26] to 162 AD patients [28], versus 214 AD patients in the present study. In addition, nutritional status was measured cross-sectional in the mentioned studies [22,26-28], while we measured weight longitudinally.

Our surprising finding that patients with more severe MTA weighed more than patients with no or mild MTA, has to be interpreted carefully since less than 10% of the patients from our cohort had a MTA score of 0 or 1. The representativeness of patients with no or mild MTA in our cohort is unclear. As expected, patients with no or mild MTA had a higher baseline MMSE score and were less dependent than patients with moderate, severe or very severe MTA [25]. Though, despite the fact that these patients had no or only mild MTA, they were referred to the memory clinic. There are no data on the weight of patients with a MTA score of 0 or 1 who were not referred to a memory clinic. It cannot be ruled out that these patients have a higher body weight than the patients with a MTA score of 0 or 1 who presented at our memory clinic.

Some limitations of the present study must be considered when interpreting the findings. Since we only investigated the relationship between MTA and body weight, it cannot be ruled out that pathology of other brain regions or other forms of brain pathology are associated with the trajectory of body weight in AD patients. In addition, our study was performed in a selected group of patients (that is they had an indication for a MRI scan and were all treated with a ChEI), which may have contributed to some degree of selection bias. ChEIs appear to slow the progression of hippocampal atrophy by 1.2% a year [51]. It cannot be ruled out that the ChEIs slowed the progression of hippocampal atrophy, thereby preventing weight loss mediated by MTA. Whether a delay of 1.2% a year is enough to prevent weight loss and even result in weight gain, is unclear. In addition, it could be that patients that lost weight were more frequently ‘lost to follow-up’ inducing a bias. However, all subjects were patients receiving care in accordance with the standardized treatment protocol, which included regular visits to our clinic. Although bias cannot fully be excluded, we think that the extent of this kind of bias is negligible since the main reason for the reduced number of subjects in our longitudinal analysis is the timing of entering the care program. In this, patients differ in the duration of participation in the care program, rather than patients leave the program due to disease-related causes. Another limitation is the absence of data regarding the trajectory of weight before the diagnosis AD was made. Weight loss may be a preclinical feature of AD [44,52]. Perhaps, weight loss in patients from our cohort may have occurred before they were referred to the memory clinic. In addition, the association between MTA and the trajectory of weight change could depend on disease severity at baseline as measured with the MMSE. Unfortunately, we did not have enough patients to stratify by MMSE or to do a sensitivity analysis. Moreover, MTA was measured cross-sectionally, instead of longitudinally. Therefore, it was not possible to investigate whether percent change in MTA over time predicts weight change, nor to elucidate whether weight loss causes disease progression by aggravating MTA. In addition, it was not possible to elucidate patterns of MTA atrophy. These patterns are, unfortunately, not fully elucidated by other studies [25]. Because of the retrospective nature of the study, we were dependent on data collected in the past. As a consequence, some data was not available, for instance information on appetite measured with a valid scale and information on dietary intake. In addition, we could not adjust for all known factors associated with weight loss, such as caregiver burden [53].

To the best of our knowledge, this is the largest study examining the relationship between MTA and the trajectory of weight change in AD patients, and the only study in which body weight was measured longitudinally. Another strength of the study is the use of the GLMM. Statistical analysis of longitudinal data is complicated because of interdependency of measurements and, particular in older AD patients, drop out of patients [54]. The GLMM is specifically developed for the analysis of longitudinal-dependent data. All data contribute to the longitudinal analysis and even data from patients who dropped out can be used. This way we could include a large number of patients with a long length of follow-up. Moreover, the severity of MTA was scored independently by two raters. The agreement between the raters was fair to good, and better than the interobserver agreement of Scheltens *et al*., which was fair [37].

## Conclusions

We found no evidence that atrophy of the medial temporal lobe is associated with weight loss in AD patients. Moreover, contrary to what was expected, AD patients did not lose but gained weight during follow-up.

## Abbreviations

AD: Alzheimer’s disease
BMI: body mass index
BPS: behavioral and psychological symptoms
CDT: clock-drawing test
CGA: comprehensive geriatric assessment
ChEIs: cholinesterase inhibitors
CIRS: cumulative illness rating scale
CT: computed tomography
FDG: [^18^F]fluordeoxyglucose
GLMM: generalized linear mixed model
MCL: Medical Center Leeuwarden
MMSE: mini mental state examination
MRI: magnetic resonance imaging
MTA: medial temporal lobe atrophy
n: number of patients
NINCDS-ADRDA: National Institute of Neurological and Communicative Diseases - Alzheimer’s Disease and Related Disorder Association
ONS: oral nutritional supplements
*P*: probability
PET: positron emission tomography
SD: standard deviation

## References

[1] Smith E, Hay P, Campbell L, Trollor JN (2011). A review of the association between obesity and cognitive function across the lifespan: implications for novel approaches to prevention and treatment. Obes Rev..

[2] Gustafson D, Rothenberg E, Blennow K, Steen B, Skoog I (2003). An 18-year follow-up of overweight and risk of Alzheimer disease. Arch Intern Med..

[3] Inelmen EM, Sergi G, Coin A, Girardi A, Manzato E (2010). An open-ended question: Alzheimer’s disease and involuntary weight loss: which comes first?. Aging Clin Exp Res..

[4] McKhann G, Drachman D, Folstein M, Katzman R, Price D, Stadlan EM (1984). Clinical diagnosis of Alzheimer’s disease: report of the NINCDS-ADRDA Work Group under the auspices of Department of Health and Human Services Task Force on Alzheimer’s Disease. Neurology..

[5] Gillette-Guyonnet S, Nourhashemi F, Andrieu S, de Glisezinski I, Ousset PJ, Riviere D (2000). Weight loss in Alzheimer disease. Am J Clin Nutr..

[6] White H, Pieper C, Schmader K, Fillenbaum G (1996). Weight change in Alzheimer’s disease. J Am Geriatr Soc..

[7] Gillette-Guyonnet S, Cortes F, Vellas B (2005). . Long-term cholinergic treatment is not associated with greater risk of weight loss during Alzheimer’s disease: data from the French REAL. FR. cohort. J Nutr Health Aging..

[8] Guérin O, Andrieu S, Schneider SM, Milano M, Boulahssass R, Brocker P (2005). Different modes of weight loss in Alzheimer disease: a prospective study of 395 patients. Am J Clin Nutr..

[9] Wolf-Klein GP, Silverstone FA, Levy AP (1992). Nutritional patterns and weight change in Alzheimer patients. Int Psychogeriatr..

[10] Besser LM, Gill DP, Monsell SE, Brenowitz W, Meranus DH, Kukull W (2014). Body mass index, weight change, and clinical progression in mild cognitive impairment and Alzheimer Disease. Alzheimer Dis Assoc Disord..

[11] Guerin O, Soto ME, Brocker P, Robert PH, Benoit M, Vellas B (2005). Nutritional status assessment during Alzheimer’s disease: results after one year (the real French study Group). J Nutr Health Aging..

[12] Vellas B, Lauque S, Gillette-Guyonnet S, Andrieu S, Cortes F, Nourhashemi F (2005). Impact of nutritional status on the evolution of Alzheimer’s disease and on response to acetylcholinesterase inhibitor treatment. J Nutr Health Aging..

[13] Andrieu S, Reynish W, Nourhashemi F, Ousset PJ, Grandjean H, Grand A (2001). Nutritional risk factors for institutional placement in Alzheimer’s disease after one year follow-up. J Nutr Health Aging..

[14] Faxen-Irving G, Basun H, Cederholm T (2005). Nutritional and cognitive relationships and long-term mortality in patients with various dementia disorders. Age Ageing..

[15] Gambassi G, Landi F, Lapane KL, Sgadari A, Mor V, Bernabei R (1999). Predictors of mortality in patients with Alzheimer’s disease living in nursing homes. J Neurol Neurosurg Psychiatry..

[16] White H, Pieper C, Schmader K (1998). The association of weight change in Alzheimer’s disease with severity of disease and mortality: a longitudinal analysis. J Am Geriatr Soc..

[17] Gillette-Guyonnet S, van Kan GA, Alix E, Andrieu S, Belmin J, Berrut G (2007). Weight loss and Alzheimer’s disease. J Nutr Health Aging..

[18] Sergi G, De Rui M, Coin A, Inelmen EM, Manzato E (2013). Weight loss and Alzheimer’s disease: temporal and aetiologic connections. Proc Nutr Soc..

[19] Aziz NA, van der Marck MA, Pijl H, Olde Rikkert MG, Bloem BR, Roos RA (2008). Weight loss in neurodegenerative disorders. J Neurol..

[20] Broberger C (2005). Brain regulation of food intake and appetite: molecules and networks. J Intern Med..

[21] Bessesen DH (2011). Regulation of body weight: what is the regulated parameter?. Physiol Behav..

[22] Grundman M, Corey-Bloom J, Jernigan T, Archibald S, Thal LJ (1996). Low body weight in Alzheimer’s disease is associated with mesial temporal cortex atrophy. Neurology..

[23] Morris CH, Hope RA, Fairburn CG (1989). Eating habits in dementia. A descriptive study. Br J Psychiatry.

[24] Macchi G, Boller F, Grafman J (1989). Anatomical substrate of emotional reactions. Handbook of neuropsychology.

[25] Whitwell JL (2010). Progression of atrophy in Alzheimer’s disease and related disorders. Neurotox Res..

[26] Hu X, Okamura N, Arai H, Higuchi M, Maruyama M, Itoh M (2002). Neuroanatomical correlates of low body weight in Alzheimer’s disease: a PET study. Prog Neuropsychopharmacol Biol Psychiatry..

[27] Burns JM, Johnson DK, Watts A, Swerdlow RH, Brooks WM (2010). Reduced lean mass in early Alzheimer disease and its association with brain atrophy. Arch Neurol..

[28] Ho AJ, Raji CA, Saharan P, DeGiorgio A, Madsen SK, Hibar DP (2011). Hippocampal volume is related to body mass index in Alzheimer’s disease. Neuroreport..

[29] Nederlandse Vereniging voor Klinische Geriatrie (NVKG). Richtlijn Comprehensive Geriatric Assessment. In: Dutch guideline regarding the comprehensive geriatric assessment. Utrecht: NVKG; 2010.

[30] Kwaliteitsinstituut voor de gezondheidszorg CBO. Richtlijn diagnostiek en medicamenteuze behandeling van dementie. In: Dutch guideline regarding the management of Alzheimer’s disease. Alphen aan den Rijn: van Zuiden Communications; 2005.

[31] National Institute for Health and Clinical Excellence (NICE). Dementia: supporting people with dementia and their carers in health and social care. In: NICE clinical guideline 42. London: NICE; 2006.

[32] Salvi F, Miller MD, Grilli A, Giorgi R, Towers AL, Morichi V (2008). A manual of guidelines to score the Modified Cumulative Illness Rating Scale and its validation in acute hospitalized elderly patients. J Am Geriatr Soc..

[33] Folstein MF, Folstein SE, McHugh PR (1975). “Mini-mental state”: a practical method for grading the cognitive state of patients for the clinician. J Pyschiatr Res..

[34] Shulman KI, Gold DP, Cohen CA, Zucchero CA (1993). Clock-drawing and dementia in the community: a longitudinal study. Int J Geriatr Psychiatry..

[35] Scheltens P, Leys D, Barkhof F, Huglo D, Weinstein HC, Vermersch P (1992). Atrophy of medial temporal lobes on MRI in “probable” Alzheimer’s disease and normal ageing: diagnostic value and neuropsychological correlates. J Neurol Neurosurg Psychiatry..

[36] Barkhof F, Fox NC, Bastos-Leite AJ, Scheltens P. Neuroimaging in dementia. Springer- Verlag: Berlin Heidelberg; 2011.

[37] Scheltens P, Launer LJ, Weinstein HC, van Gool WA (1995). Visual assessment of medial temporal lobe atrophy on magnetic resonance imaging: interobserver reliability. J Neurol..

[38] Scheltens P, van der Pol L (2012). Atrophy of medial temporal lobes on MRI in “probable” Alzheimer’s disease and normal ageing: diagnostic value and neuropsychological correlates. J Neurol Neurosurg Psychiatry.

[39] Cohen J (1960). A coefficient of agreement for nominal scales. Educ Psychol Meas..

[40] Fitzmaurice GM, Ravichandran C (2008). A primer in longitudinal data analysis. Circulation..

[41] Breslow NE, Clayton DG (1993). Approximate inference in generalized linear mixed models. J Am Stat Assoc..

[42] Landis JR, Koch GG (1977). The measurement of observer agreement for categorical data. Biometrics..

[43] Secher M, Andrieu S, Gillette-Guyonnet S, Soto M, Rolland Y, Cantet C (2013). Weight changes in Alzheimer’s disease patients with increased aberrant motor behavior. Int J Geriatr Psychiatry..

[44] Gu Y, Scarmeas N, Cosentino S, Brandt J, Albert M, Blacker D (2014). Change in body mass index before and after Alzheimer’s disease onset. Curr Alzheimer Res..

[45] Gillette-Guyonnet S, Andrieu S, Cortes F, Nourhashemi F, Cantet C, Ousset PJ (2006). Outcome of Alzheimer’s disease: potential impact of cholinesterase inhibitors. J Gerontol A Biol Sci Med Sci..

[46] Guerin O, Andrieu S, Schneider SM, Cortes F, Cantet C, Gillette-Guyonnet S (2009). Characteristics of Alzheimer’s disease patients with a rapid weight loss during a six-year follow-up. Clin Nutr..

[47] Mets T, De Deyn PP, Pals P, De Lepeleire J, Vandewoude M, Ventura M (2013). COGNOS: care for people with cognitive dysfunction: a national observational study. Alzheimer Dis Assoc Disord..

[48] Gustafson D, Lissner L, Bengtsson C, Björkeland C, Skoog I (2004). A 24-year follow-up of body mass index and cerebral atrophy. Neurology..

[49] Ward MA, Carlsson CM, Trivedi MA, Sager MA, Johnson SC (2005). The effect of body mass index on global brain volume in middle-aged adults: a cross sectional study. BMC Neurol..

[50] Gustafson DR, Luchsinger J (2013). High adiposity: risk factor for dementia and Alzheimer’s disease?. Alzheimers Res Ther..

[51] Hashimoto M, Kazui H, Matsumoto K, Nakano Y, Yasuda M, Mori E (2005). Does donepezil treatment slow the progression of hippocampal atrophy in patients with Alzheimer’s disease?. Am J Psychiatry..

[52] Johnson DK, Wilkins CH, Morris JC (2006). Accelerated weight loss may precede diagnosis in Alzheimer disease. Arch Neurol..

[53] Bilotta C, Bergamaschini L, Arienti R, Spreafico S, Vergani C (2010). Caregiver burden as a short-term predictor of weight loss in older outpatients suffering from mild to moderate Alzheimer’s disease; a three months follow-up study. Aging Ment Health..

[54] Mohs R, Schmeidler J, Aryan M (2000). Longitudinal studies of cognitive, functional and behavioural change in patients with Alzheimer’s disease. Stat Med..

